# Endogenous Galectin-8 protects against Th17 infiltration and fibrosis following acute kidney injury

**DOI:** 10.1186/s10020-025-01245-y

**Published:** 2025-05-16

**Authors:** Elisa Perez-Moreno, Adely de la Peña, Tomás Toledo, Javiera Saez, Francisca Pérez-Molina, Sofía Espinoza, Claudia Metz, Nicole Díaz-Valdivia, Lorena Azócar, Carolina Prado, Rodrigo Pacheco, Fabian Segovia-Miranda, Alejandro S. Godoy, Cristian A. Amador, Teo Feuerhake, Alfonso González, Andrea Soza

**Affiliations:** 1 Centro de Biología Celular y Biomedicina (CEBICEM), Facultad de Ciencias, Universidad San Sebastián, Santiago, Chile; 2 Centro Científico Tecnológico de Excelencia Ciencia y Vida, Fundación Ciencia y Vida, Santiago, Chile; 3 Laboratorio de Fisiopatología Renal, Facultad de Ciencias, Universidad San Sebastián, Santiago, Chile; 4 Laboratorio de Neuroinmunología, Facultad de Ciencias, Universidad San Sebastián, Santiago, Chile; 5 Department of Cell Biology, Faculty of Biological Sciences, Universidad de Concepción, Concepción, Chile; 6 Department of Pathology, School of Medicine, Pontificia Universidad Católica de Chile, Santiago, Chile; 7 Facultad de Medicina, Universidad San Sebastián, Santiago, Chile

**Keywords:** Galectin-8, Acute kidney injury, AKI, Fibrosis, Chronic kidney disease, CDK, Inflammation and Th17

## Abstract

**Background:**

Acute kidney injury (AKI) is a serious clinical condition characterized by a rapid decline in renal function, often progressing to chronic kidney disease (CKD) and fibrosis. The endogenous mechanisms influencing kidney injury resolution or maladaptive repair remain poorly understood. Galectin-8 (Gal-8), a tandem-repeat β-galactoside-binding lectin, plays a role in epithelial cell proliferation, epithelial-mesenchymal transition, and immune regulation, all of which are critical in AKI outcomes. While exogenous Gal-8 administration has shown renoprotective effects, its endogenous role in kidney injury progression and resolution remains unclear.

**Methods:**

To investigate the endogenous role of Gal-8 in AKI, we compared the responses of Gal-8 knockout (Gal-8-KO; *Lgals8*^*−/−*^ bearing a β-gal cassette under the Lgals8 gene promoter) and wild-type (*Lgals8*^+*/*+^) mice in a nephrotoxic folic acid (FA)-induced AKI model. Renal Gal-8 expression was assessed by β-galactosidase staining, lectin-marker colocalization, and RT-qPCR. Renal function, structure, and immune responses were evaluated at the acute (day 2) and fibrotic (day 14) phases of injury. Plasma creatinine levels were measured to assess renal function, while histological analyses evaluated tubular damage, renal inflammation, and extracellular matrix deposition. Flow cytometry was performed to characterize the immune response, focusing on pro-inflammatory T cells.

**Results:**

Galectin-8 was predominantly expressed in the renal cortex, localizing to tubules, glomeruli, and blood vessels, with its levels decreasing by half following AKI. Both *Lgals8*^+*/*+^ and *Lgals8*^*−/−*^ mice exhibited similar renal function and structure impairments during the acute phase, though *Lgals8*^+*/*+^ mice showed slightly worse damage. By the fibrotic phase, *Lgals8*^*−/−*^ mice exhibited more pronounced cortical damage and fibrosis, characterized by increased type I and III collagen deposition and enhanced Th17 cell infiltration, while myofibroblast activation remained comparable to that of *Lgals8*^+*/*+^ mice.

**Conclusions:**

Endogenous Gal-8 does not significantly protect the kidney during the acute phase and is dispensable for cell proliferation and death in response to AKI. However, it is crucial in preventing maladaptive repair by regulating extracellular matrix homeostasis and mitigating fibrosis. Additionally, Gal-8 contributes to inflammation resolution by limiting persistent immune cell infiltration, particularly IL-17-secreting cells.

## Background

Acute kidney injury (AKI) is a clinical condition marked by a rapid decline in renal function, often triggered by ischemia, nephrotoxic compounds, or sepsis (Basile et al. 2012; Kellum et al. 2021). AKI remains a significant global health concern, affecting approximately 13 million people each year and resulting in nearly 2 million deaths worldwide (Zuk and Bonventre 2016). Despite its prevalence, treatment options are limited, and AKI often progresses to chronic kidney disease (CKD) and kidney failure (Basile et al. 2001; Basile 2004; Coca et al. 2012; Moeller et al. 2021; Panizo et al. 2021; Guo et al. 2023; Niculae et al. 2023). A deeper understanding of the molecular mechanisms underlying AKI progression is critical for developing targeted therapies that could promote kidney repair and prevent fibrosis.

Following AKI, tubular epithelial cell death led to tissue damage and reduced renal function (Sancho-Martinez et al. 2015; Martin-Sanchez et al. 2017; Guo et al. 2023; Kolbrink et al. 2023). Surviving cells dedifferentiate and transiently undergo EMT to promote regeneration (Humphreys et al. 2008; Chang-Panesso and Humphreys 2017; Lee et al. 2021). Fibroblasts and pericytes are also temporarily activated, contributing to tissue remodeling. However, when injury is severe or repetitive, repair becomes maladaptive, resulting in fibrosis and progression to CKD (Basile et al. 2001; Basile 2004; Coca et al. 2012; LeBleu et al. 2013; Ferenbach and Bonventre 2015; Humphreys 2018; Moeller et al. 2021; Panizo et al. 2021; Guo et al. 2023; Niculae et al. 2023).

The immune system plays a critical role in the development of kidney fibrosis (Sato and Yanagita 2018; Fu et al. 2022; Marino et al. 2023). Following kidney injury, immune cells such as macrophages, neutrophils, and dendritic cells are activated, releasing pro-inflammatory cytokines and chemokines that recruit additional immune cells to the injury site (Chen et al. 2018; Singbartl et al. 2019). Under normal conditions, the immune response resolves through anti-inflammatory signals and regulatory immune cells, such as T regulatory cells (Tregs), which promote cellular debris clearance and tissue repair (Serhan and Savill 2005; Chen et al. 2018). However, unresolved inflammation in maladaptive responses exacerbates tissue damage and drives progressive kidney function loss (Chung et al. 2018). A key factor in the pathological immune response in kidney injury is the increased activity of Th17 cells, a subtype of CD4 + T helper cells (Chan et al. 2014; Mehrotra et al. 2015). Persistent Th17 cell activity favors the progression from AKI to CKD (Burne et al. 2001; Mehrotra et al. 2015), while neutralizing its activity reduces fibrosis (Pechman et al. 2008; Basile et al. 2021). Identifying endogenous factors that prevent exaggerated Th17-mediated inflammation following AKI could reveal potential therapies aimed at mitigating maladaptive repair and renal fibrosis.

Galectin-8 (Gal-8) is one of the most widely expressed members of the galectin protein family in human tissues (Elola et al. 2014), with diverse cellular functions that may influence AKI outcomes (Perez-Moreno et al. 2024a, 2024b). Its effects may complement or counteract those of other galectins implicated in inflammation and fibrosis (Marino et al. 2023). As a glycan-binding protein of the tandem-repeat type of galectins, Gal-8 possesses two carbohydrate recognition domains (CRDs) separated by a linker peptide of variable length, each exhibiting distinct carbohydrate-binding preferences (Ideo et al. 2011; Cagnoni et al. 2020). Its N-terminal CRD exhibits affinity for α−2,3 sialylated and 3′-sulfated oligosaccharides, unique among galectins, while its C-terminal CRD preferentially binds non-sialylated oligosaccharides, including blood antigens and poly-N-acetyl-lactosamine (Ideo et al. 2011; Cagnoni et al. 2020).

Like other galectins, Gal-8 is synthesized in the cytosol and secreted through unconventional pathways, possibly involving exosomes (Popa et al. 2018). Intracellularly, it plays a crucial role in cellular quality control and innate defense against pathogens by detecting glycans exposed on the luminal side of damaged endosomes and lysosomes, thereby promoting their autophagic removal (Thurston et al. 2012; Boyle and Randow 2013; Falcon et al. 2018; Bell et al. 2021). Once secreted, extracellular Gal-8 interact with a variety of cell surface glycoproteins with signaling receptor functions, integrins and ECM components (Levy et al. 2001; Carcamo et al. 2006; Norambuena et al. 2009; Vicuna et al. 2013; Sampson et al. 2016; Oyanadel et al. 2018; Prato et al. 2020; Zick 2022). Through these interactions, Gal-8 may modulate diverse cellular processes that influence adaptive or maladaptive tissue repair following AKI, including epithelial cell proliferation, survival and EMT (Oyanadel et al. 2018; Perez-Moreno et al. 2024b), as well as immune responses that shape inflammation (Pardo et al. 2017; Obino et al. 2018; Prato et al. 2020; Zick 2022; Prato et al. 2024).

In the immune system, Gal-8 influences the T cell repertoire (Tribulatti et al. 2007) and facilitates adaptive immune responses by promoting B cell differentiation (Tsai et al. 2011; Anginot et al. 2013), antigen presentation, and T cell activation (Tribulatti et al. 2009; Cattaneo et al. 2011; Obino et al. 2018). Additionally, Gal-8 can either amplify or restrict inflammatory responses by regulating interactions between leukocytes, platelets, and endothelial cells (Cattaneo et al. 2014; Toegel et al. 2014; Weinmann et al. 2018; Tribulatti et al. 2020). Notably, Gal-8 exhibits immunosuppressive properties by inducing Tregs (Sampson et al. 2015, 2016; Pardo et al. 2017) and triggering apoptosis of activated Th17 cells (Norambuena et al. 2009; Sampson et al. 2015, 2016; Pardo et al. 2017). Given that the intensity of Th17 cell activity is crucial in the outcome of AKI (Burne et al. 2001; Pechman et al. 2008; Mehrotra et al. 2015; Basile et al. 2021), Gal-8 may serve as a key regulator of inflammatory processes after renal damage.

Interestingly, the immunoregulatory functions of Gal-8 can be disrupted by function-blocking anti-Gal-8 autoantibodies, which have been detected in autoimmune and inflammatory conditions, including sepsis (Carcamo et al. 2006; Massardo et al. 2009; Vicuna et al. 2013; Pardo et al. 2017, 2019). This suggests that Gal-8 dysfunction could exacerbate immune-mediated tissue damage in AKI. Given its reported actions on epithelial cell biology and the immune system, it is essential to define not only its therapeutic potential but also its endogenous role in AKI outcomes. We previously demonstrated that exogenous administration of Gal-8 protects kidneys from AKI and prevents the expression of fibrosis-related genes (Perez-Moreno et al. 2024b). In this study, we investigate the endogenous role of Gal-8 in renal damage by using a *LgalS8*^*−/−*^ mice, which lack Gal-8 expression, in a nephrotoxic model of FA-induced AKI. By assessing its impact on kidney function, fibrosis and immune response during both the acute and fibrotic phases after AKI, we aim to elucidate whether Gal-8 plays a protective role in kidney injury resolution or contributes to maladaptive repair.

## Methods

### Animals and mouse model of acute kidney injury

Animal experiments were approved by the Ethical Committee of Universidad San Sebastián (Protocol number 01–2021-10). Female C57BL/6 NTac mice were maintained in standard cages under constant temperature (21ºC) and in 12:12 light–dark cycle, with free access to tap water and food. Lgals8/Lac-Z knock-in (here called *Lgals8*^*−/−*^) mice were engineered in Regeneron Pharmaceuticals Inc., New York, using Velocigene technology, as previously described (Pardo et al. 2019). *Lgals8*^*−/−*^ mice have Lac-Z gene under the control of Gal-8 promotor, expressing β-galactosidase in replace to Gal-8. *Lgals8*^+*/*+^ and *Lgals8*^*−/−*^ mice were genotyped using PCR, and 16 weeks old mice were selected for experiments. AKI was induced as described (Yan 2021) in *Lgals8*^+*/*+^ and *Lgals8*^*−/−*^ mice. The control group (*n* = 5) received 200 μl of sodium bicarbonate 0,3 M. To induce AKI, mice were injected with a single dose of 250 mg/ml of folic acid (FA) (*n* = 5). For their analysis, kidneys were collected 2 and 14 days after sodium bicarbonate or FA injection and were divided for freezing or fixation in 4% formaldehyde.

### Renal function analysis

Creatinine was measured at the end of each experiment to assess the renal function. Blood samples were taken from the vena cava and plasma was isolated by centrifugation at 8000 rpm for 15 min. Creatinine was measured using a colorimetric assay (Wiener Lab) following the manufacturer’s instructions.

### β-galactosidase staining in *Lgals8*^*−/−*^mice

Due to the lack of specific antibodies for detecting Gal-8 in mice, we used Gal-8 knockout/LacZ knock-in mice (*Lgals8*^*−*^*/*^*−*^) to detect endogenous Gal-8 expression. In this model, the entire Gal-8 gene (*Lgals8*) is replaced with a β-galactosidase (β-gal) reporter cassette under the control of the Gal-8 promoter, allowing indirect detection of Gal-8 via β-gal staining (Pardo et al. 2017, 2019). *Lgals8*^*−/−*^ mice were perfused with paraformaldehyde 4% prior to kidney collection. Kidneys were fixed in paraformaldehyde 4%, and maintained in sucrose 30% for 48 h until they were frozen in Tissue-Tek® O.C.T. compound (Sakura Finetek). Six μm sections were obtained using a Leica cryostat. β-galactosidase stain was performed as described (Pardo et al. 2017). Biotinilated lectins LTA (1:400, Vector Labs B-1325–2) and DBA (1:400, Vector Labs B-1035–5) were used to stain convoluted proximal tubules and collecting ducts, respectively. Biotinilated lectin WGA (1:400, Vector Labs B-1025–5) was used to stain the whole nephron. Lectins were detected using Alexa Fluor-568 conjugated Streptavidin (ThermoFisher). Images were obtained using a Leica SP8 scanning confocal microscope.

### Histopathology

The kidneys were fixed in a 4% buffered formalin solution, dehydrated and embedded in paraffin. Hematoxylin–eosin (H&E), Masson’s trichrome, Sirius Red and Periodic acid-Schiff (PAS) staining were performed in 4μm tissue sections, and microscopic examination of renal tissue was blindly evaluated by a pathologist (T.F.). The total renal cortex area and the extent of acute tubular injury across all cortical foci were measured in μm^2^ using ImageJ software. Acute cortical tubular damage was graded based on the percentage of affected area as follows: no significant damage (< 10%), mild (10–25%), moderate (25–50%), and severe damage (> 50%). PAS staining was used to determine tubular dilation, measuring the diameter of 100 tubules per sample using ImageJ. Slides stained with Masson's Trichrome and Sirius Red were scanned with a digital slide scanner (Aperio AT2, Leica Biosystems) for digital pathology analysis at 20 × magnification. Images were analyzed using Aperio ImageScope software (Leica Biosystems) to quantify fibrosis and collagen-positive areas in kidney sections.

### Immunofluorescence and immunohistochemistry in formalin-fixed paraffin-embedded kidneys

Immunofluorescence (IF) and immunohistochemistry (IHC) in 4 μm tissue sections were performed as described (Perez-Moreno et al. 2024b) using the following primary antibodies: Ki67 (1:400, Cell Signaling #12,202), α-SMA (1:400, DAKO M0851). For IF, incubation of Alexa Fluor-647 secondary antibody was performed at room temperature and images were obtained using a Leica SP8 scanning confocal microscope. The fluorescence intensity of α-SMA in the renal interstitium and the number of Ki67-positive nuclei were quantified using ImageJ.

### Detection of apoptosis in kidney sections

Apoptosis was detected in formalin fixed-paraffin embedded kidney sections using DeadEnd™ Fluorometric TUNEL System (Promega, G3250), following manufacturer’s guidance. Images were obtained in a Leica SP8 scanning confocal microscope. Ten random images per sample were taken for their analysis and TUNEL-positive nuclei were counted using ImageJ software.

### RNA extraction and RT-qPCR

Total RNA extraction was performed from frozen tissue using Trizol (Invitrogen). Reverse transcription was performed as previously described (Norambuena et al. 2009), using the following primers:

5’ TGAACACCAATGCCCGAAGC 3’ (Forward) and 5’ GCGTGGGTTCAAGTGCAGAG 3’ (Reverse) for Gal-8; 5’ ATGCCCATCTTCTGCTTGTCA 3’ (Forward) and 5’ CCTTGTAGTTGTGGGTCTTGT 3’ (Reverse) for KIM-1; 5’ AACTGCATCTGCCCTAAGGTCTTC 3’ (Forward) and 5’ TAAGGCATCACAGTCCGAGTCACA 3’ (Reverse) for MCP-1; 5’ TTATGGCTCAGGGTCCAACTCTGT 3’ (Forward) and 5’ GCAGAACTCAGGAATGGACATTCG 3’ (Reverse) for TNF-α; 5’ TACAGTGAAGGCAGCAGCGATCA 3’ (Forward) and 5’ TGACGTGGAACGGTTGAGGTAGTC 3’ (Reverse) for IL-17; and 5’ AGTGTGACGTTGACATCCGT 3’ (Forward) and 5’ GCAGCTCAGTAACAGTCCGC 3’ (Reverse) for β-actin. The expression levels of the genes of interest were normalized relative to the levels of β-actin gene expression.

### Protein extraction and western blot

Kidneys were homogenized in lysis buffer with protease and phosphatase inhibitors as described (Perez-Moreno et al. 2024b). Western blotting was performed with 50 μg of protein, using the following primary antibodies: NGAL (Abcam ab63929), α-SMA (DAKO M0851), vimentin (Cell Signaling, #5741), Fibronectin-1 (Santa Cruz Biotechnology, sc-271098) and GAPDH (Cell Signaling, #5174). Anti-rabbit IgG (Rockland, N° 611–1322) and anti-mouse IgG (Rockland, N° 610–1302) secondary antibodies were incubated 1 h at RT. Densitometric analyses were carried out using ImageJ Software.

### Isolation of kidney mononuclear cells for flow cytometry analysis

The organ was dissected, and tissue was minced into small pieces and digested by collagenase D (2.5 mg/mL; Roche Diagnostics) and DNase I (1 mg/mL; Sigma) at 37 °C for 45 min. Digested tissue was filtered through a 70 µm cell strainer obtaining a single cell suspension that was subjected to centrifugation in a density gradient made with Percoll (70%/30%). Mononuclear cells were removed from the interphase and resuspended in culture medium for further analysis. Fluorochrome-conjugated monoclonal antibodies (mAb) specific to mouse CD45 (clone 30-F11), CD4 (clone GK-1.5), IFNγ (clone XMG1.2), IL-17 (clone TC11-18H10.1), TCRβ (clone B183983) and CD8α (clone 58–6,7) were purchased from Biolegend. For analysis of cytokine production, cells were re-stimulated with 1 μg/mL ionomycin (Sigma) and 50 ng/mL PMA (Sigma) in the presence of 5 μg/mL brefeldin A (Invitrogen) for 3 h before immunostaining. For intracellular immunostaining, cells were first labeled with antibodies specific for cell-surface markers and then fixed and permeabilized with FoxP3 Fixation/Permeabilization kit (eBioscience). Afterward, cytokine immunostaining was performed in permeabilized cells, followed by flow cytometry analysis. All analyses assessed live/dead discrimination using Zombie Aqua (ZAq) Fixable Viability kit (Biolegend). Data were collected with a FACSCanto II (BD) and results were analyzed with FACSDiva (BD) and FlowJo software (Tree Star).

### Statistical analysis

Results are shown as mean ± SEM. Statistical analysis was performed using GraphPad Prism version 8.0. An unpaired t-test was performed to compare two experimental groups. Comparisons between *LgalS8*^+*/*+^ and *LgalS8*^*−/−*^ mice were performed using the two-way ANOVA followed by Sidak’s multiple comparisons test. *P*-value < 0.05 was considered statistically significant.

## Results

### Kidney expression of Gal-8

**Fig. 1:**
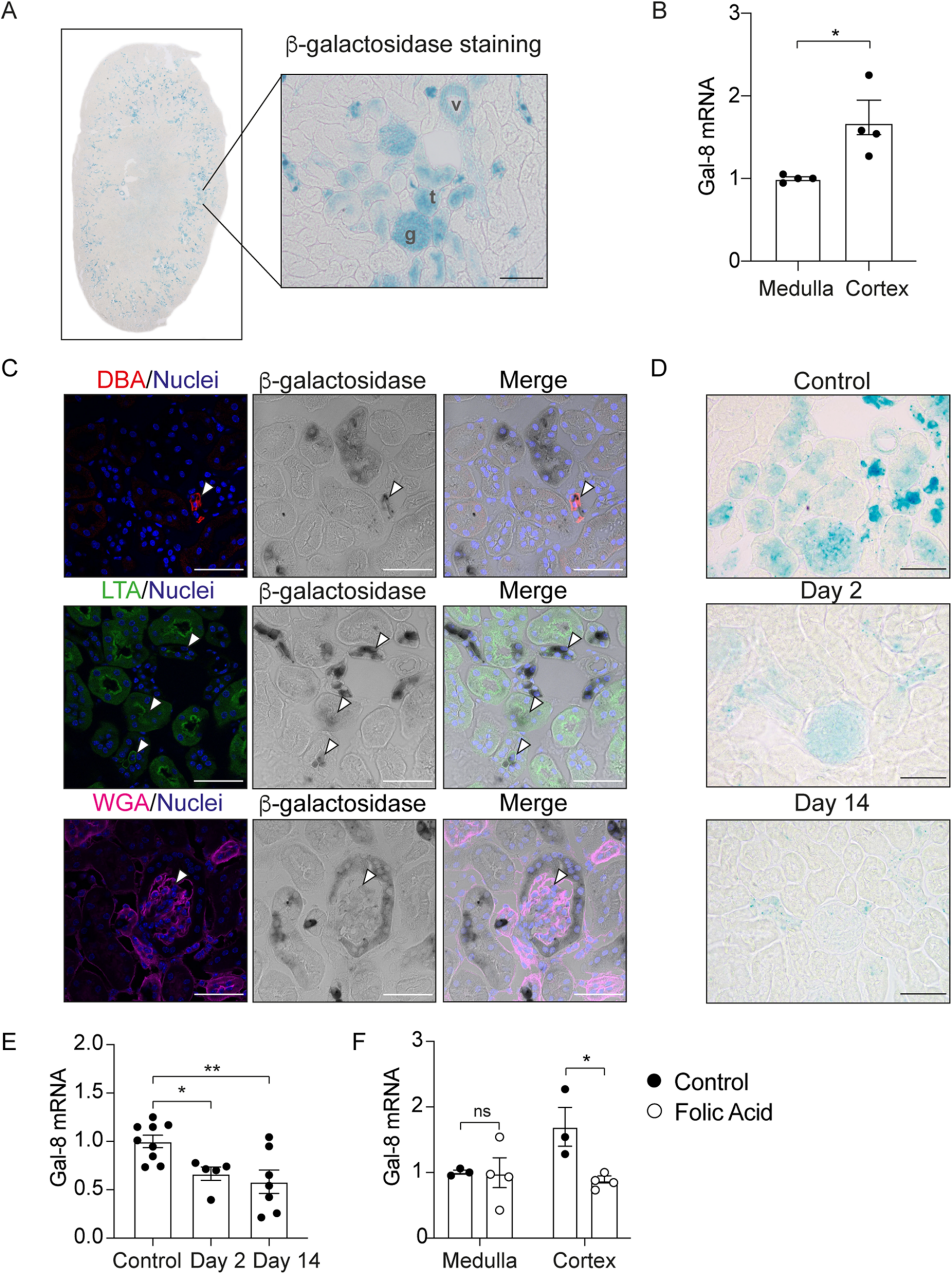
Gal-8 is expressed in the kidney and its levels decrease following AKI. **A** β-galactosidase staining in kidneys from *Lgals8*^*−/−*^ mice, reflecting endogenous Gal-8 expression. The inset shows a magnified view of the renal cortex. g = glomeruli, t = tubule, v = blood vessel. Scale bar: 100 μm. **B** Gal-8 transcript levels in the renal medulla and cortex of *Lgals8*^+*/*+^ mice, measured by RT-qPCR and normalized to β-actin. **C** Lectin staining of kidney structures in *Lgals8*^*−/−*^ mice: collecting ducts (DBA, red), proximal tubules (LTA, green), and whole kidney (WGA, magenta). β-galactosidase staining is shown in grayscale. Arrows indicate colocalization of lectin and β-galactosidase staining. Scale bar: 50 μm. **D** β-galactosidase staining in kidneys from *Lgals8*^*−/−*^ mice at 2 and 14 days after AKI induction. Scale bar: 50 μm. **E** Gal-8 transcript levels in kidneys from *Lgals8*^+*/*+^ mice at 2 and 14 days after AKI induction, assessed by RT-qPCR and normalized to β-actin. **F** Gal-8 transcript levels in the renal medulla and cortex of *Lgals8*^+*/*+^ mice at 2 days post-AKI induction, measured by RT-qPCR and normalized to β-actin. Data is presented as mean ± SEM of *n* = 3–9. *p < 0.05, **p < 0.01

To explore whether endogenous Gal-8 is protective against kidney injury as described for exogenously administered Gal-8 (Perez-Moreno et al. 2024b), it is first essential to determine whether and where this lectin is expressed in this organ. Unfortunately, anti-Gal-8 antibodies, either commercially available or generated in our laboratory (Pardo et al. 2017, 2019), gave positive staining in the tissues of Gal-8 knock-out mice and, therefore, are unsuitable for immunohistochemistry. Our previously described approach takes advantage of the Gal-8 knock out/LacZ knock-in mice (*Lgals8*^*−/−*^*)*, in which the entire Gal-8 gene (*LGals8*) has been replaced by a β-galactosidase (β -gal) reporter cassette under the Gal-8 promoter (Pardo et al. 2017, 2019). Examining the kidney of *Lgals8*^*−/−*^ mice we found β-galactosidase staining reflecting the activity of the Gal-8 promoter mainly in the renal cortex and including tubules (t), glomeruli (g), and blood vessels (v) (Fig. 1A). Consistent with this data, the kidneys of *Lgals8*^+*/*+^ mice showed higher Gal-8 mRNA levels in the renal cortex compared to the medulla (Fig. 1B).

In addition, different lectins can be used to identify specific glycan structures within the kidney (Laitinen et al. 1987). For instance, DBA (Dolichos biflorus agglutinin) binds to N-acetylgalactosamine (GalNAc) and marks collecting ducts, LTA (Lotus tetragonolobus agglutinin) recognizes fucose residues and primarily stains the proximal tubules, whereas WGA (Wheat germ agglutinin) detects N-acetylglucosamine (GlcNAc) and sialic acid, serving as a marker for glomeruli and glycan-rich structures (Laitinen et al. 1987). The kidney of *Lgals8*^*−/−*^ mice displayed β-galactosidase staining colocalizing with DBA, LTA, and WGA staining, thus corroborating that Gal-8 is expressed in glomeruli and tubules, being predominant in collecting ducts (Fig. 1C).

### Gal-8 expression in the kidney decreases after FA-induced AKI

The widely-studied model of FA-induced renal damage allows for distinguishing tissular events within an acute phase of 1–3 days and a fibrotic phase occurring beyond 7 days (Feng et al. 2019; Yan 2021). We applied the β-galactosidase staining and the quantitative mRNA approach to evaluate whether AKI changes the expression of Gal-8. FA-induced damage resulted in reduced β-galactosidase staining in *Lgals8*^*−/−*^ mice measured during the acute phase after 2 days and the fibrotic phase at day 14 (Fig. 1D). Similarly, we detected lower levels of Gal-8 mRNA in the kidneys of *Lgals8*^+*/*+^ mice, decreasing ~ 50%, both 2 and 14 days after AKI induction (Fig. 1E). Separating the medulla from the cortex we found that such decrease affected specifically the renal cortex, where FA exerts the damage (Yan 2021), while the medulla did not change (Fig. 1F). These results indicate that Gal-8 expression in the cortex decreases almost half during AKI and remains low throughout the fibrotic phase.

### Differences in tubular injury between *Lgals8*^+*/*+^*and Lgals8*^*−/−*^ mice during the acute phase of renal injury

**Fig. 2:**
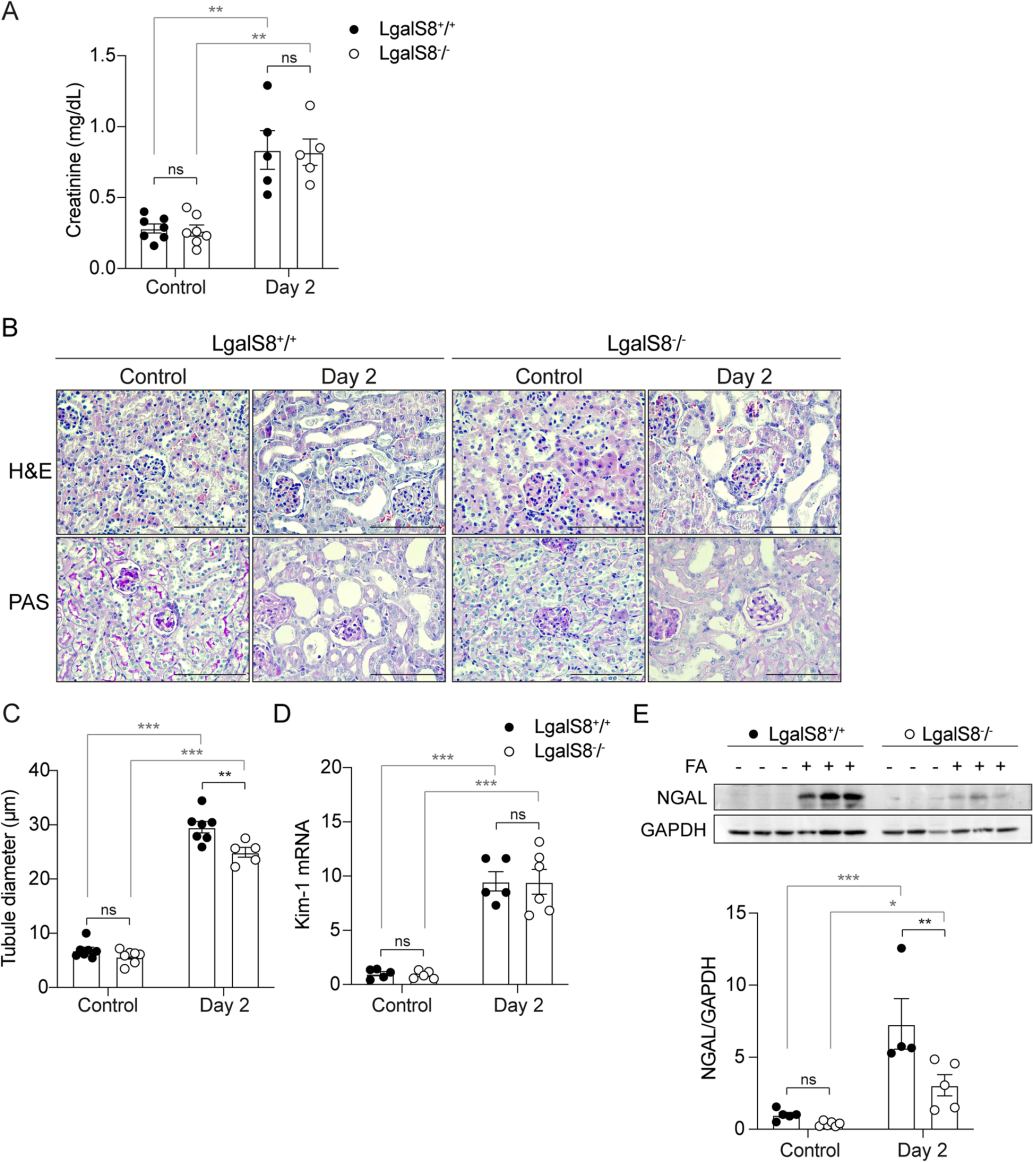
Renal impairment during the acute phase after AKI in *Lgals8*^*−/−*^ and *Lgals8*^+*/*+^ mice. **A** Plasma creatinine levels measured 2 days after AKI induction in *Lgals8*^+*/*+^ and *Lgals8*^*−/−*^ mice. **B** H&E and PAS staining in renal cortex from *Lgals8*^+*/*+^ and *Lgals8*^*−/−*^ mice. Representative images captured at 40X magnification. Scale bar: 100 μm. **C** Tubular dilation quantified in histological sections from *Lgals8*^+*/*+^ and *Lgals8*^*−/−*^ mice. **D** Transcript levels of the tubular injury marker KIM-1, determined by RT-qPCR and normalized to β-actin. **E** Immunoblot quantification of NGAL protein levels in *Lgals8*^+*/*+^ and *Lgals8*^*−/−*^ mice, normalized to GAPDH. Mean ± SEM of *n* = 4–7. *p < 0.05, **p < 0.01, ***p < 0.001

To assess whether endogenous Gal-8 affects the outcome of AKI, we compared parameters associated with kidney damage, including renal function, tubular dilation, and tubular injury markers in *Lgals8*^+*/*+^ and *Lgals8*^*−/−*^ mice during the acute phase. Creatinine levels increased significantly and similarly in both *Lgals8*^+*/*+^ and *Lgals8*^*−/−*^ mice 2 days after AKI induction, indicating a deterioration of renal function (Fig. 2A). H&E and PAS staining reveal tubular dilation, epithelial flattening, brush border loss, and luminal debris accumulation in *Lgals8*^+*/*+^ and *Lgals8*^*−/−*^ mice (Fig. 2B). Tubular dilation was significantly greater in *Lgals8*^+*/*+^ mice compared to *Lgals8*^*−/−*^* mice* during the acute phase (Fig. 2C).

To further analyze the extent of tubular injury, we assessed acute tubular damage markers such as KIM-1 (Kidney Injury Molecule-1) and NGAL (neutrophil gelatinase-associated lipocalin) (Vaidya et al. 2008). Transcript levels of KIM-1 increased similarly in both *Lgals8*^+*/*+^ and *Lgals8*^*−/−*^ mice (Fig. 2D). NGAL protein levels also increased in both *Lgals8*^+*/*+^ and *Lgals8*^*−/−*^ mice after 2 days, reaching higher levels in the *Lgals8*^+*/*+^ mice (Fig. 2E). The observed discrepancy may be explained by the type of measurement (protein vs. mRNA) and the distinct segmental expression patterns of NGAL and KIM-1 within the nephron. Whereas KIM-1 is expressed in proximal tubules during AKI, NGAL is detected in proximal and distal tubule in response to renal damage (Latoch et al. 2020).

Taken together, these functional, histological, and molecular evaluations point to slightly worse damage suffered by the kidneys of *Lgals8*^+*/*+^ mice, suggesting that endogenous Gal-8 does not significantly protects kidneys during the acute phase.

### Proliferation and cell death after AKI are not affected by the lack of Gal-8 expression

**Fig. 3:**
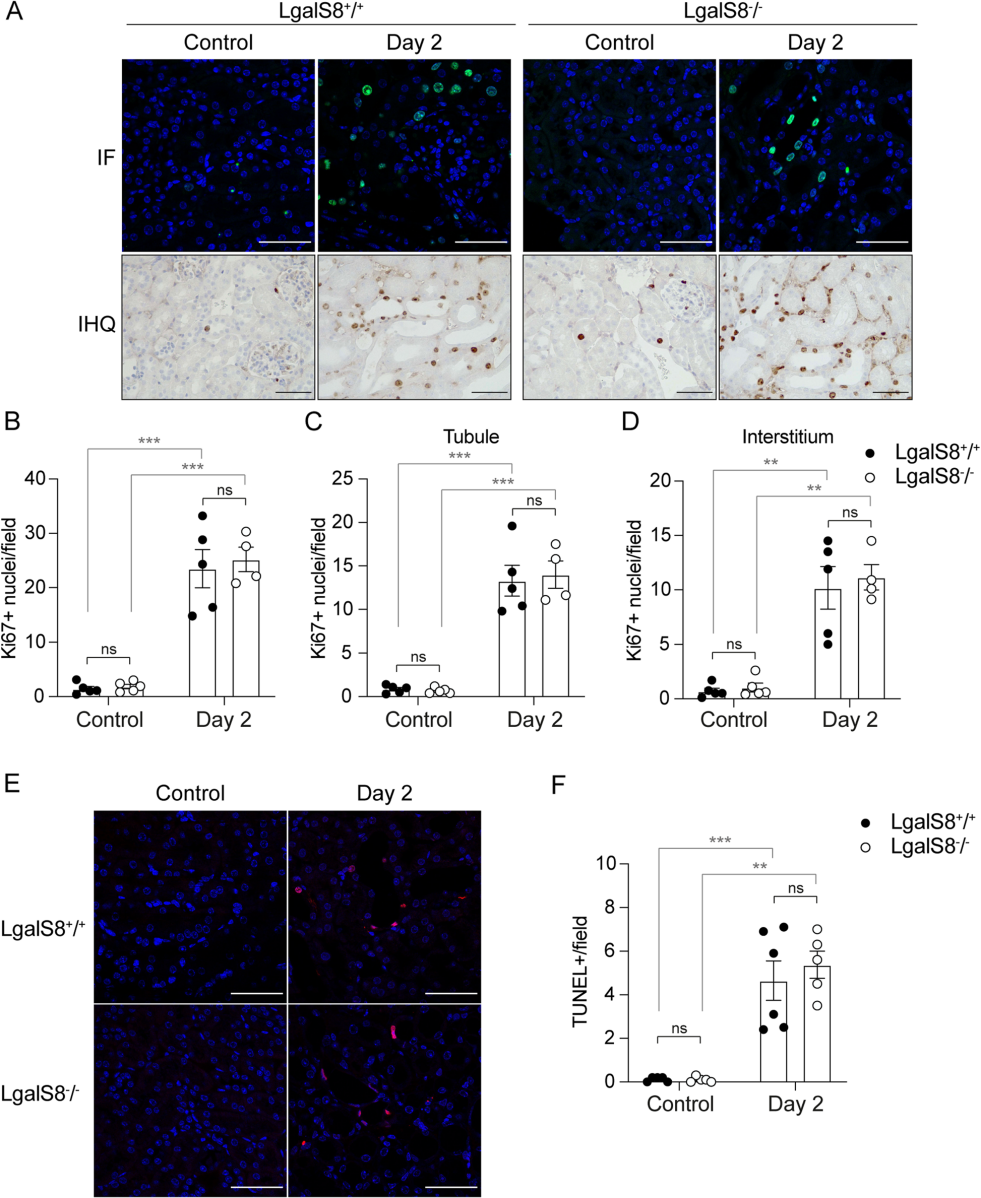
Similar levels of cell proliferation and death during the acute phase of AKI in *Lgals8*^*−/−*^ and *Lgals8*^+*/*+^ mice. **A** Immunofluorescence (IF) and immunohistochemistry (IHQ) for the proliferation marker Ki67 assessed 2 days after AKI induction in *Lgals8*^+*/*+^ and *Lgals8*^*−/−*^ mice. Scale bar: 50 μm. **B** Quantification of total Ki67-positive nuclei. **C** Quantification of Ki67-positive cells in tubular epithelial and (**D**) interstitial cells of *Lgals8*^+*/*+^ and *Lgals8*^*−/−*^ mice. **E** Cell death assessment by TUNEL assay in kidney sections from *Lgals8*^+*/*+^ and *Lgals8*^*−/−*^ mice, 2 days after AKI induction. Scale bar 50 μm. **F** Quantification of TUNEL-positive cells. Data is presented as mean ± SEM of *n* = 4–6. **p < 0.01, ***p < 0.005

Repair of the renal epithelium after damage begins when surviving cells proliferate to replace dead cells and restore renal function (Yang et al. 2010). To assess whether endogenous Gal-8 contributes to the proliferative response following kidney damage, we evaluated the proliferation marker Ki67 in histological sections (Fig. 3A). Immunofluorescence analysis of Ki67 revealed a similar increase in cell proliferation in *Lgals8*^+*/*+^ and *Lgals8*^*−/−*^ mice two days after AKI induction (Fig. 3B). Immunohistochemistry for Ki67 demonstrated that proliferation occurred in both tubular and interstitial cells (Fig. 3A). A detailed analysis comparing proliferating tubular and interstitial cells showed no significant differences between *Lgals8*^+*/*+^ and *Lgals8*^*−/−*^ mice during the acute phase of injury (Fig. 3C, D). In addition, we did not observe significant alterations in cell proliferation in the glomeruli (data not shown).

FA promotes cell death mainly in proximal tubular epithelial cells (Yan 2021). To analyze the effect of Gal-8 on FA-induced cell death, we compared TUNEL assay on histological sections of *Lgals8*^+*/*+^ and *Lgals8*^*−/−*^ mice (Fig. 3E). Cell death increased similarly in *Lgals8*^+*/*+^ and *Lgals8*^*−/−*^ mice at day 2 (Fig. 3F). These findings suggest that endogenous Gal-8 is dispensable for the overall rate of cell proliferation and death in response to AKI.

### FA-induced renal damage leads to higher cortical damage and collagen-associated fibrosis in *Lgals8*^*−/−*^ mice

**Fig. 4:**
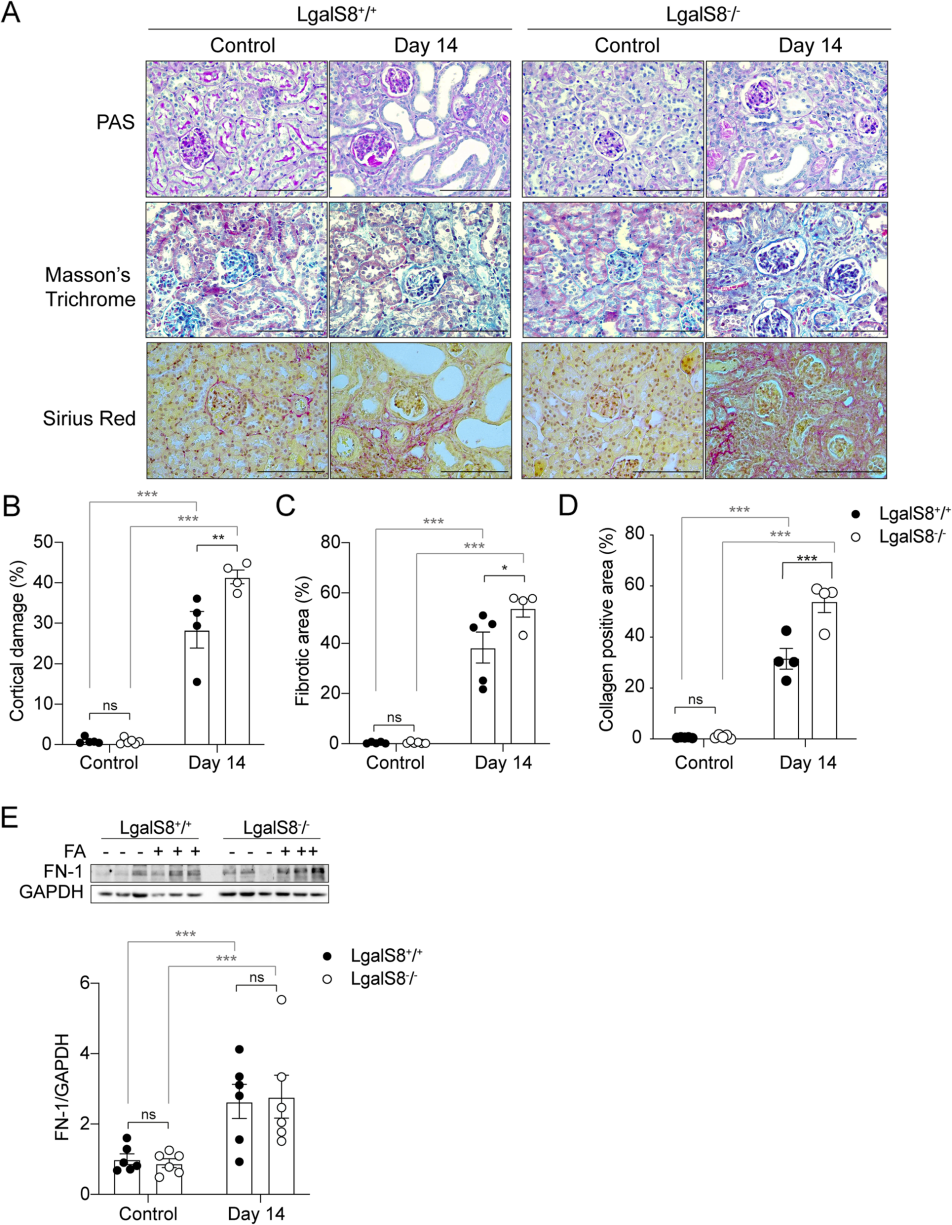
Cortical damage and fibrosis are increased during the fibrotic phase in *Lgals8*^*−/−*^ mice. **A** PAS, Masson´s Trichrome and Sirius Red staining in representative images of kidney sections from *Lgals8*^+*/*+^ and *Lgals8*^*−/−*^ mice 14 days after damage induction. Scale bar: 100 μm. **B** Renal cortical damage measurements show the extent of the injured area in *Lgals8*^+*/*+^ and *Lgals8*^*−/−*^ mice. **C** Fibrotic area quantified by collagen stained blue in kidney sections from *Lgals8*^+*/*+^ and *Lgals8*^*−/−*^ mice. **D** Collagen-positive area was quantified by collagen stained red in kidney sections. **E** Immunoblot quantification of fibronectin-1 in the kidney of *Lgals8*^+*/*+^ and *Lgals8*^*−/−*^ mice normalized to GAPDH. Data represent mean ± SEM of *n* = 4–6, *p < 0.05, **p < 0.01, ***p < 0.001

Then, we analyzed the cortical damage during the fibrotic phase 14 days after FA administration. PAS staining used to detect global structural loss in the cortical area revealed higher damage in the *Lgals8*^*−/−*^ mice (Fig. 4A, B). On the other hand, interstitial fibrosis is a hallmark of maladaptive repair associated with poor prognosis due to disruption of tissue architecture and irreversible decline in renal function (Ferenbach and Bonventre 2015; Moeller et al. 2021; Panizo et al. 2021). This pathological process is characterized by the excessive accumulation of extracellular matrix, which results in scarring and impaired kidney function (LeBleu et al. 2013; Moeller et al. 2021; Panizo et al. 2021). Therefore, we compared *Lgals8*^+*/*+^ and *Lgals8*^*−/−*^ mice kidney sections stained for matrix protein, mainly collagen, after 14 days post-injury (Fig. 4A). Figure 4A shows representative images of Masson’s Trichrome staining detecting mainly collagen and other matrix proteins and Sirius Red staining for Type-I and III collagen deposition, characteristics of renal damage. Remarkably, *Lgals8*^*−/−*^ mice showed a significantly higher fibrotic area than *Lgals8*^+*/*+^ mice (Fig. 4C, D), which accompanied the enhanced cortical damage shown in Fig. 4B for this genotype.

To complement the histological analysis, we assessed the levels of fibronectin-1, another fibrosis-related protein (Fig. 4E). *Lgals8*^+*/*+^ and *Lgals8*^*−/−*^ mice showed similarly increased levels of fibronectin-1 protein, measured 14 days after AKI (Fig. 4E). All these results suggest that endogenous Gal-8 plays a crucial role in the extracellular matrix homeostasis of the kidney, particularly collagen, contributing to ameliorating the potential fibrotic outcome and maladaptive repair after AKI.

### Gal-8 expression does not influence myofibroblast activation

**Fig. 5:**
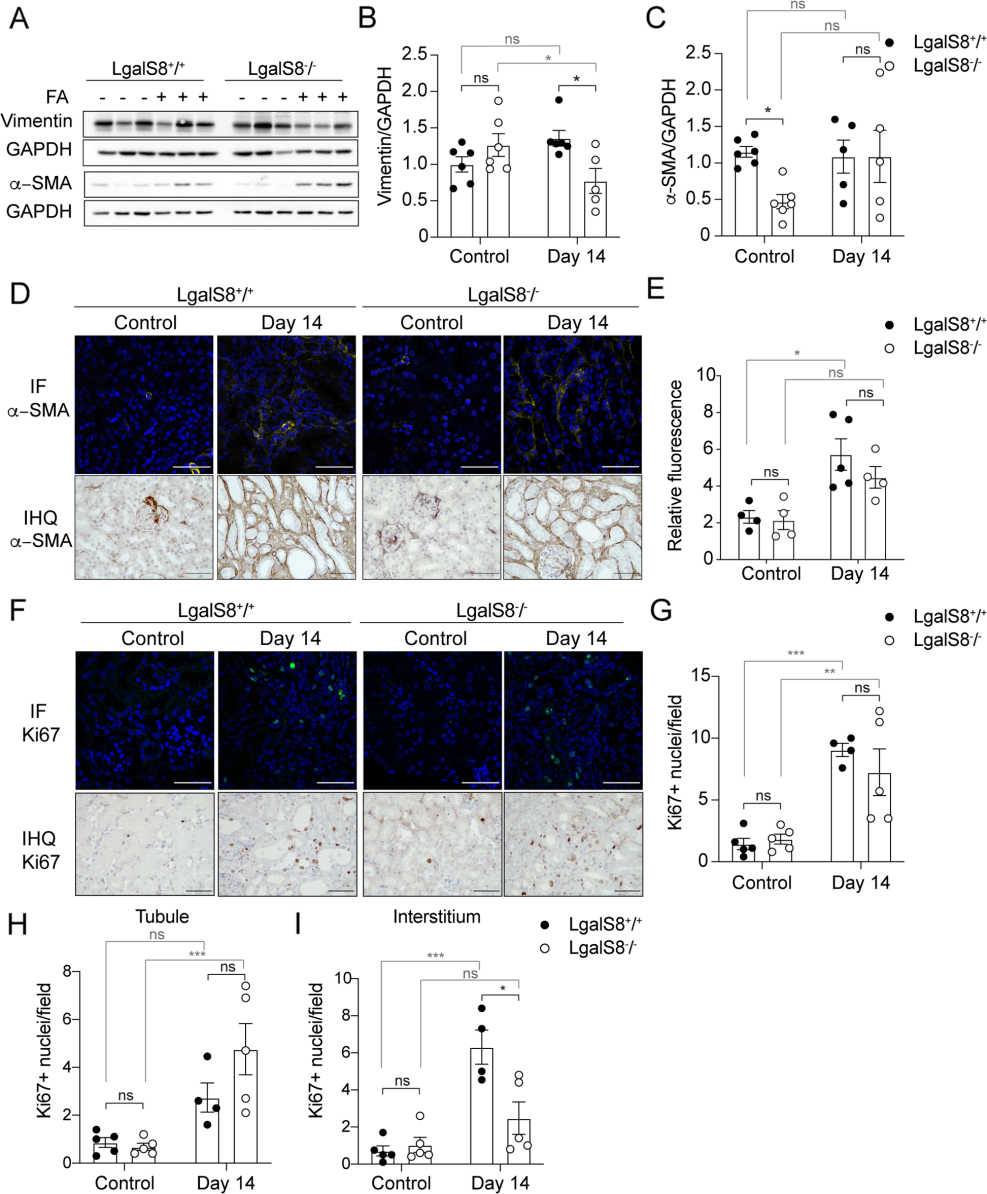
Similar fibroblast activation in the fibrotic phase in *Lgal8*^+*/*+^ and *Lgals8*^*−/−*^ mice. (**A**) Vimentin and α-SMA protein levels compared by immunoblot 14 days after AKI induction in *Lgals8*^+*/*+^ and *Lgals8*^*−/*−^ mice, normalized to GAPDH. (**B**) and (**C**) graphs quantifying the relative levels of vimentin and α-SMA, respectively. **D** Immunofluorescence (IF) and immunohistochemistry (IHQ) representative images of α-SMA localization in the kidney of *Lgals8*^+*/*+^ and *Lgals8*^*−/*−^ mice. Scale bar: 50 μm. **E** Quantification of fluorescence intensity in α-SMA IF staining. **F** Cell proliferation activity shown by Ki67 IF and IHQ staining. **G** Quantification of total Ki67-positive nuclei. **H** Ki67-positive cells in tubular epithelial and (**I**) interstitial cells of *Lgals8*^+*/*+^ and *Lgals8*^*−/*−^ mice. Data is presented as mean ± SEM of *n* = 4–6. *p < 0.05, **p < 0.01, ***p < 0.001

Activated myofibroblasts are the primary contributors to the excessive extracellular matrix (ECM) accumulation in renal fibrosis (Walker et al. 2018; Djudjaj and Boor 2019). Therefore, we assessed fibroblast activation by measuring the protein levels of mesenchymal markers such as vimentin and α-SMA (Fig. 5A). By day 14 after injury induction, *Lgals8*^*+/+*^ and *Lgals8*^*−/−*^ mice showed similar levels of vimentin and α-SMA (Fig. 5B,C), even though *Lgals8*^*−/−*^ mice showed only half of the basal levels of α-SMA (Fig. 5A,C). Contrary to western blot, immunofluorescence and immunohistochemistry of α-SMA showed only interstitial staining with similar levels and distribution among both genotypes (Fig. 5D,E). This difference may be attributed to the methodology employed, as western blots assess protein levels across the entire tissue, whereas IF quantification specifically measures α-SMA within the interstitium. The observed differences between *Lgal8*^*+/+*^ and *Lgals8*^*−/−*^ mice may originate from sources other than the interstitium.

Myofibroblasts are interstitial cells that become activated and proliferate to promote fibrosis (Zhou and Liu 2016). Here, we observe that the lack of endogenous Gal-8 does not affect the total number of proliferating cells but reduces cell proliferation in the renal interstitium compared to *Lgals8*^+*/*+^ mice without significantly affecting tubular cell proliferation (Fig. 5F-I). The results suggest that myofibroblast activity alone is not enough to explain the higher fibrotic outcome observed in *Lgals8*^−/−^ mice in Fig. 4. Other cells present in the interstitium likely contribute to these results.

### Enhanced Infiltration of Th17 and Tc17 cells after renal injury in *Lgals8*^*−/−*^ mice

We previously described that exogenously added Gal-8 acts as an immunosuppressant by inducing apoptosis of Th17 cells in a mice model of experimental autoimmune encephalomyelitis (Pardo et al. 2017). IL-17 is a pro-inflammatory cytokine contributing to tissue damage and fibrosis in AKI and CKD (Basile et al. 2021).

**Fig.6:**
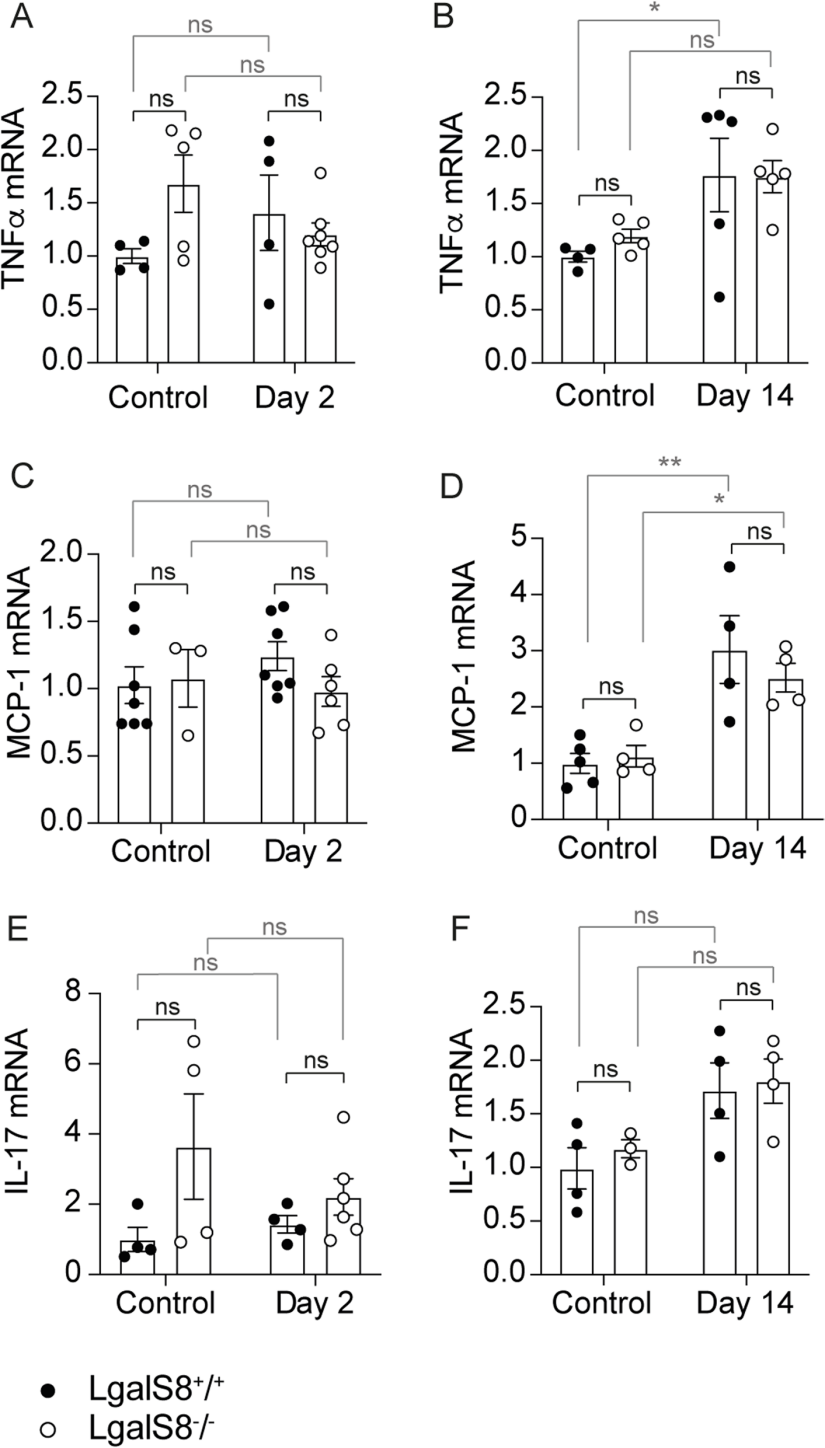
Expression of pro-inflammatory markers in *Lgal8*^+*/*+^ and *Lgals8*^*−/−*^ mice. mRNA levels of TNF-α (**A**-**B**), MCP-1 (**C**-**D**), and IL-17 (**E**–**F**) were measured at 2 and 14 days after injury induction in *Lgals8*^+*/*+^ and *Lgals8*^*−/*−^ mice by RT-qPCR and normalized to β-actin. Data is presented as mean ± SEM of *n* = 4–7. *p < 0.05, **p < 0.01

To investigate whether endogenous Gal-8 plays an immunomodulatory role in renal damage, we measured mRNA levels of the pro-inflammatory cytokines TNF-α, MCP-1 and IL-17 in the kidneys 2 and 14 days after damage induction (Fig. 6). During the acute phase of injury, no significant differences were observed between *Lgals8*^+*/*+^ and *Lgals8*^*−/−*^ mice for any of the cytokines analyzed (Fig. 6A,C,E). At day 14 post-injury, *Lgals8*^+*/*+^ mice presented a significant increase in TNF-α mRNA levels (Fig. 6B). *Lgals8*^*−/−*^ also presented an increase in TNF-α expression, although not statistically significant (Fig. 6B). MCP-1 levels were elevated in both *Lgals8*^+*/*+^ and *Lgals8*^*−/−*^ mice (Fig. 6D), while IL-17 exhibited a slight, non-significant increase in both genotypes (Fig. 6F). These results suggest an increased inflammation during the fibrotic phase of kidney damage. While Gal-8 may not strongly influence acute-phase cytokine expression, it may participate in modulating the inflammatory milieu during later stages of tissue response.

**Fig.7:**
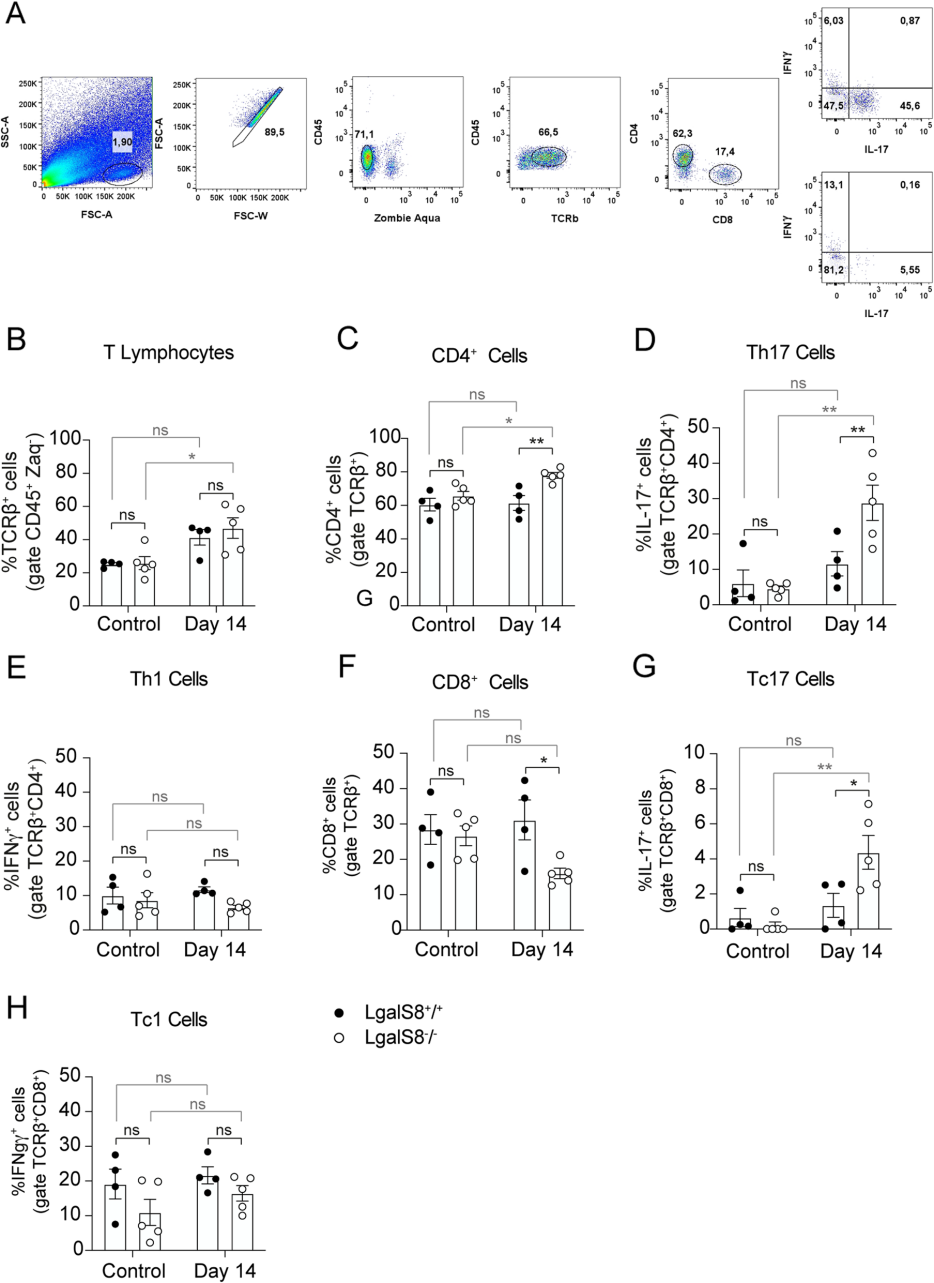
Enhanced Th17 kidney infiltration in *Lgals8*^*−/−*^ mice. **A** A gating strategy was used to analyze cytokine production on stimulated CD4 and CD8 T cells from kidneys. Lymphocytes were gated based on size and granularity, and then doublet exclusion was performed. Events from these gates were plotted against Zombie Aqua viability dye and CD45 marker. Cells were further selected for CD45 and TCRβ positivity before CD4 and CD8 gating was applied. Finally, IFNγ and IL-17 production were evaluated in CD4^+^ and CD8^+^ populations. Fourteen days after damage induction, mononuclear cells from kidneys were isolated and analyzed by flow cytometry to detect the infiltration of (**A**) total T cells, (**B**) CD4^+^ T cells, (**C**) Th1 cells, (**D**) Th17 cells, (**E**) CD8^+^ T cells, (**F**) Tc1 cells and (**G**) Tc17 cells in *Lgals8*^+*/*+^ and *Lgals8*^*−/−*^ mice. Mean ± SEM of *n* = 4–5. *p < 0.05, **p < 0.005

To further understand the immunomodulatory effect of Gal-8 on kidney damage, we performed flow cytometry during the fibrotic phase of injury (Fig. 7). The Gating strategy is shown in Fig. 7A. Flow cytometry of lymphocyte subpopulations at 14 days post-injury revealed similar TCRβ^+^ lymphocyte infiltration in *Lgals8*^+*/*+^ and *Lgals8*^*−/−*^ mice. However, *Lgals8*^*−/−*^ mice exhibited increased infiltration compared to the control condition (Fig. 6B). The infiltration of TCRβ^+^CD4^+^ T cells was increased 14 days after AKI induction in *Lgals8*^*−/−*^ mice relative to *Lgals8*^+*/*+^ (Fig. 6C). Notably, the percentage of a subset of TCRβ^+^CD4^+^ T cells, the Th17 cells, was significantly higher in *Lgals8*^*−/−*^ mice than in *Lgals8*^+*/*+^ mice under damage conditions (Fig. 6D).

Although, *Lgals8*^*−/−*^ mice also showed reduced TCRβ^+^CD8^+^ T cells kidney infiltration (Fig. 6F), they displayed an increased percentage of Tc17 cells (Fig. 6G), a subset of CD8^+^ T cells that produce IL-17 (Liang et al. 2015). In contrast, Tc1 lymphocytes, which produce interferon-γ showed no significant differences among *Lgals8*^+*/*+^ and *Lgals8*^*−/−*^ mice (Fig. 6H). These results suggest that endogenous Gal-8 is essential in resolving inflammation by limiting the persistence of immune cell infiltration, particularly IL-17-secreting cells, after kidney injury.

## Discussion

This study provides evidence that endogenous Galectin-8 (Gal-8) mitigates kidney inflammation and fibrosis by reducing the infiltration of Th17 lymphocytes following an acute kidney injury (AKI) episode.

Folic acid (FA)-induced renal damage serves as a well-established model for studying tissue events during acute and fibrotic phases, with the acute phase occurring within the first 3 days and the fibrotic phase beyond 7 days after FA administration (Feng et al. 2019; Yan 2021). During the acute phase, cell death is followed by a recovery phase characterized by the proliferation and redifferentiation of surviving epithelial cells, thus reflecting tubular repair mechanisms (Ishibe and Cantley 2008). The fibrotic phase involves the activation and proliferation of interstitial fibroblasts, which play a key role in extracellular matrix (ECM) deposition (Yan 2021). To explore the role of endogenous Gal-8 in these dynamics, we compared kidney functional and structural parameters during the acute (2 days) and fibrotic (14 days) phases in FA-treated *Lgals8*^+*/*+^ and *Lgals8*^*−/−*^ mice.

In the acute phase, we observed no significant differences in renal function between mice with or without endogenous Gal-8. However, in the *Lgals8*^+*/*+^ mice, tubular dilation and the molecular marker of injury NGAL displayed significantly higher levels, which might initially suggest that Gal-8 negatively influences the early phase of kidney damage. A more likely possibility arises from our findings that Gal-8 is expressed in the renal cortex, particularly in glomeruli and epithelial cells of the collecting ducts and proximal tubules, key sites of FA-induced damage (Yan 2021). We found that Gal-8 expression decreases by nearly half following AKI. Our previous study demonstrated that exogenous Gal-8 administration confers a protective effect following FA-induced AKI (Perez-Moreno et al. 2024b), suggesting that the decreased endogenous Gal-8 expression observed here could be mitigated by exogenous Gal-8 treatment. Why, then, *Lgals8*^+*/*+^ mice exhibit slightly more significant tubular damage during the acute phase following AKI induction? One possibility is that the constitutive absence of Gal-8 in *Lgals8*^*−/−*^ mice triggers compensatory mechanisms that reduce damage, whereas the acute downregulation of endogenous Gal-8 in *Lgals8*^+*/*+^ mice, might result in the exacerbation of early AKI outcomes unless supplemented with exogenous Gal-8.

Maladaptive repair leading to fibrosis involves a cascade of events that promote inflammation and recurrent damage, ultimately resulting in tubular loss and a progressive decline in kidney function (Ferenbach and Bonventre 2015). These events include epithelial cell injury and death, vascular rarefaction, persistent partial epithelial-mesenchymal transition (pEMT), premature cellular senescence, G2/M cell-cycle arrest, activation of fibroblasts and the recruitment of immune cells (Ferenbach and Bonventre 2015). Fibroblast activation and ECM deposition are key drivers of kidney fibrosis, significantly increasing the risk of developing a chronic kidney disease (CKD) (Yang et al. 2010; Lovisa et al. 2015; Sheng and Zhuang 2020; Lee et al. 2021; Rayego-Mateos et al. 2022). In our previous study, we demonstrated the anti-fibrotic effects of exogenously administered Gal-8 following FA-induced AKI (Perez-Moreno et al. 2024b). Gal-8 treatment aids renal tubular epithelial cells in overcoming cell cycle arrest and promotes their re-differentiation in response to nephrotoxic AKI (Perez-Moreno et al. 2024b). Exogenously added Gal-8 has been shown to promote cell proliferation in various contexts, including glioblastoma cells (Metz et al. 2016), keratinocytes (Lo et al. 2021), T cells (Cattaneo et al. 2011) and MDCK cells (Oyanadel et al. 2018). In this study, the analysis of *Lgals8*^+*/*+^ and *Lgals8*^*−/−*^ mice suggests that endogenous Gal-8 does not protect against tubular cell death and does not promote tubule cell proliferation, as both parameters were similarly increased in the two genotypes. However, endogenous Gal-8 seems to promote interstitial cell proliferation during the fibrotic phase 14 days after FA administration. These findings indicate that while endogenous Gal-8 does not affect the overall rate of cell proliferation, its absence impairs cellular responses within the interstitial compartment during the fibrotic phase of the injury.

Our results with PAS staining indicate that endogenous Gal-8 protects against global structural loss in the cortical area. Additionally, the analysis of collagen and other matrix proteins by Masson’s Trichrome staining, along with the assessment of collagen I and III by Sirius Red staining, reveal that endogenous Gal-8 mitigates fibrosis development. These findings highlight the protective role of Gal-8 in preventing maladaptive repair following AKI.

Upregulation of collagen I and III is an early event in the progression toward renal fibrosis, with collagen I as a key structural component of fibrotic tissue (Bulow and Boor 2019). The renal ECM is a dynamic structure that undergoes continuous remodeling, particularly during fibrosis. An imbalance in collagen turnover inevitably disrupts tissue function, underscoring the critical role of collagen degradation in maintaining tissue homeostasis (Bulow and Boor 2019; Sprangers and Everts 2019). Fibrillar collagen is primarily degraded by matrix metalloproteinases (MMPs), including MMP-1, MMP-2, MMP-8, MMP-13, MMP-14 (known as MT1-MMP) and MMP-16 (MT3-MMP) (McKleroy et al. 2013; Sprangers and Everts 2019). Notably, Gal-8 has been shown to increase MMP-2 and MMP-9 protein levels in trophoblasts (Legner et al. 2024), MMP-13 activity in MDCK cells (Oyanadel et al. 2018), and the secretion of MMP-1, MMP-3 and MMP-13 in chondrocytes (Weinmann et al. 2018). It is possible that the reduction in collagen deposition observed in *Lgals8*^+/+^ mice may be attributed to changes in MMP activity mediated by Gal-8. The activation and proliferation of interstitial fibroblasts have been implicated in the fibrotic phase of the FA-induced AKI model (Yan 2021). Myofibroblasts, the primary source of ECM proteins, can originate from various sources, including resident fibroblasts, bone marrow-derived fibroblasts, endothelial cells, pericytes or even macrophages (Abu El-Asrar et al. 2011; LeBleu et al. 2013; Falke et al. 2015; Kanisicak et al. 2016; Meng et al. 2016; Yuan et al. 2019). However, our data indicated that fibroblast activation following injury is similar in *Lgals8*^+*/*+^ and *Lgals8*^*−/−*^ mice. Thus, the increased fibrosis observed in *Lgals8*^*−/−*^ mice cannot be fully explained by fibroblast activation alone. We observed a dramatic increase in Th17 cell infiltration in *Lgals8*^*−/−*^ mice. IL-17 contributes to fibrosis by activating various cell types beyond fibroblasts, fostering an inflammatory and pro-fibrotic environment that promotes maladaptive repair (Sisto and Lisi 2023).

Gal-8 functions as an anti-inflammatory agent in various contexts, including infections (Bertelli et al. 2020) and autoimmune disorders (Sampson et al. 2015, 2016; Pardo et al. 2017). Its mechanisms of action involve the induction of apoptosis of activated Th17 cells and differentiation of regulatory T cells (Tregs) (Sampson et al. 2015, 2016; Pardo et al. 2017). In experimental autoimmune encephalomyelitis (EAE), a widely-used animal model of multiple sclerosis (MS), the absence of Gal-8 exacerbates severity, leading to enhanced inflammation, extensive demyelination, and elevated levels of pro-inflammatory Th17 cells. Notably, exogenous Gal-8 administration effectively counteracts these effects, significantly alleviating symptoms by inducing apoptosis of activated Th17 cells (Pardo et al. 2017). Here, we observed that *Lgals8*^*−/−*^ mice exhibited enhanced inflammation characterized by increased infiltration of IL-17-producing Th17 and Tc17 cells. Both subpopulations are implicated in renal responses to injury, contributing to fibrosis and chronic inflammation (Burne et al. 2001; Liang et al. 2015; Mehrotra et al. 2018). Th17 cells are mediators of kidney fibrosis, representing the most abundant kidney-infiltrating lymphocytes following AKI in mice and rats (Chan et al. 2014; Mehrotra et al. 2015, 2017; Rabb et al. 2016; Dellepiane et al. 2020; Basile et al. 2021). Inhibition of Th17 lymphocytes with mycophenolate mofetil (MMF) reduces the progression of renal fibrosis in post-AKI rats exposed to a high-salt diet (Mehrotra et al. 2015), highlighting its relevance to kidney fibrosis. We found increased infiltration of Th17 cells in *Lgals8*^*−/−*^ mice during the fibrotic phase after AKI. Therefore, the immunomodulatory role of Gal-8 most likely contributes to a protective role that counteracts the fibrotic outcome after renal damage.

Our results show that Gal-8 expression decreases after AKI and remains lower for at least 14 days, contrasting with other galectins, such as Gal-1, Gal-3, and Gal-9, which have been reported to increase during injury (Vansthertem et al. 2010; Kolatsi-Joannou et al. 2011; Prud'homme et al. 2019; Volarevic et al. 2019; Kulow et al. 2024). Among these, Gal-3 is the most studied and has been implicated in either protective (Volarevic et al. 2019; Kulow et al. 2024) or pro-fibrotic effects after kidney damage (Henderson et al. 2008; Kolatsi-Joannou et al. 2011; Martinez-Martinez et al. 2016; Li et al. 2018; Volarevic et al. 2019; Hermenean et al. 2022; Marino et al. 2023; Perez-Moreno et al. 2024a), depending on the model studied. Similar to our findings, increased renal damage in Gal-3 KO mice has been linked to an inflammatory phenotype characterized by elevated infiltration of Th1 and Th17 lymphocytes (Volarevic et al. 2019). Elevated serum Gal-3 levels are associated with an increased risk of developing CKD in individuals from the Framingham Heart Study Cohort (Kannel et al. 1979; O'Seaghdha et al. 2013), and are inversely correlated with the estimated glomerular filtration rate (eGFR) in patients with chronic heart failure (Tang et al. 2011). Furthermore, serum Gal-3 levels are elevated in patients with sepsis and post-cardiac surgery patients who develop AKI (Sun et al. 2021a, 2021b). These observations underscore the involvement of endogenous Gal-3 in the onset and progression of renal damage. However, there is currently no information on the patient’s serum Gal-8 levels after AKI episodes. Investigating these parameters could help establish Gal-8 as a potential biomarker for assessing the risk of maladaptive repair following AKI.

Could other pathogenic conditions mimic the *Lgals8*^*−/−*^ mice by reducing the protective endogenous role of Gal-8? An intriguing possibility is the presence of function-blocking anti-Gal-8 autoantibodies. These autoantibodies have been detected in association with leucopenia in systemic lupus erythematosus (Massardo et al. 2009; Pardo et al. 2019), and as biomarkers of worse prognosis in relapsing–remitting forms of multiple sclerosis (Pardo et al. 2017). Additionally, patients with rheumatoid arthritis or sepsis also generate anti-Gal-8 autoantibodies, implicating autoimmunity and severe inflammatory conditions as general causative factors (Massardo et al. 2009; Pardo et al. 2019). These findings raise concerns about the progression of AKI in patients who may develop Gal-8 autoantibodies, particularly in sepsis, where around two-thirds of patients with septic shock experience AKI (Manrique-Caballero et al. 2021). Monitoring serum levels of Gal-8 and anti-Gal-8 autoantibodies could be valuable biomarkers to assess the risk of developing more severe AKI or fibrosis, particularly in high-risk patients.

## Conclusions

Our findings demonstrate that Gal-8 is expressed in proximal convoluted tubules and collecting ducts, with its levels significantly decreasing following AKI. We show that endogenous Gal-8 plays a protective role in nephrotoxic kidney damage by exerting anti-fibrotic and anti-inflammatory effects involving down-regulation of Th17 cells.

## Data Availability

No datasets were generated or analysed during the current study.

## Abbreviations

AKI: Acute kidney injury
CKD: Chronic kidney disease
CRD: Carbohydrate recognition domain
EAU: Experimental autoimmune uveitis
ECM: Extracellular matrix
EMT: Epithelial-mesenchymal transition
IRI: Ischemia-reperfusion injury
KIM-1: Kidney injury molecule-1
FA: Folic acid
MCP: Modified citrus pectin
NGAL: Neutrophil gelatinase-associated lipocalin
UUO: Unilateral ureteral obstruction
